# EQUIP Healthcare: An overview of a multi-component intervention to enhance equity-oriented care in primary health care settings

**DOI:** 10.1186/s12939-015-0271-y

**Published:** 2015-12-14

**Authors:** Annette J. Browne, Colleen Varcoe, Marilyn Ford-Gilboe, C. Nadine Wathen, on behalf of the EQUIP Research Team

**Affiliations:** School of Nursing, The University of British Columbia, T201 – 2211 Wesbrook Mall, Vancouver, BC V6T 2B5 Canada; Arthur Labatt Family School of Nursing, Western University, H37 Health Sciences Addition, 1151 Richmond St., London, ON N6A 5C1 Canada; Faculty of Information & Media Studies, Western University, North Campus Building, Room 240, 1151 Richmond St., London, ON N6A 5B7 Canada

**Keywords:** Health equity, Health inequities, Intervention research, Case study, Trauma- and violence-informed care, Cultural safety, Primary health care, Primary care, Indigenous populations, Marginalized populations, Structural violence

## Abstract

**Background:**

The primary health care (PHC) sector is increasingly relevant as a site for population health interventions, particularly in relation to marginalized groups, where the greatest gains in health status can be achieved. The purpose of this paper is to provide an overview of an innovative multi-component, organizational-level intervention designed to enhance the capacity of PHC clinics to provide equity-oriented care, particularly for marginalized populations. The intervention, known as EQUIP, is being implemented in Canada in four diverse PHC clinics serving populations who are impacted by structural inequities. These PHC clinics serve as case studies for the implementation and evaluation of the EQUIP intervention. We discuss the evidence and theory that provide the basis for the intervention, describe the intervention components, and discuss the methods used to evaluate the implementation and impact of the intervention in diverse contexts.

**Design and methods:**

Research and theory related to equity-oriented care, and complexity theory, are central to the design of the EQUIP intervention. The intervention aims to enhance capacity for equity-oriented care at the staff level, and at the organizational level (i.e., policy and operations) and is novel in its dual focus on:

- Staff education: using standardized educational models and integration strategies to enhance staff knowledge, attitudes and practices related to equity-oriented care in general, and cultural safety, and trauma- and violence-informed care in particular, and;
- Organizational integration and tailoring: using a participatory approach, practice facilitation, and catalyst grants to foster shifts in organizational structures, practices and policies to enhance the capacity to deliver equity-oriented care, improve processes of care, and shift key client outcomes.

Using a mixed methods, multiple case-study design, we are examining the impact of the intervention in enhancing staff knowledge, attitudes and practices; improving processes of care; shifting organizational policies and structures; and improving selected client outcomes.

**Discussion:**

The multiple case study design provides an ideal opportunity to study the contextual factors shaping the implementation, uptake and impact of our tailored intervention within diverse PHC settings. The EQUIP intervention illustrates the complexities involved in enhancing the PHC sector's capacity to provide equity-oriented care in real world clinical contexts.

## Background: Why enhance capacity for equity-oriented PHC services?

Research shows that the primary health care^1^ (PHC) sector is increasingly relevant as a site for population health interventions, particularly in relation to marginalized 2 groups, where the greatest gains in health status can be achieved [1–8]. Broad-based PHC interventions – that integrate accessible, high quality services with structural and/or policy changes to improve people’s access to the social determinants of health -- may be one of the most effective means of achieving health equity for marginalized populations [1, 9]. However, few such interventions have been developed and tested, particularly in the Canadian context.

Despite Canada’s national health care program, health and health care inequities are increasing in the context of oppressive neoliberal health and social policies [10–12]. Health inequities can be understood as socially constructed, unjust, and avoidable differences in health and well-being between and within groups of people [13]. These inequities structure patterns of individual ill health and population-level morbidity and mortality rates [14]. Equity in health is, therefore, a social justice goal focused on pursuing the highest possible standard of health and health care for all people, paying special attention to those at greatest risk of poor health, and taking into account broader socio-political and economic influences on health and access to care [14, 15].

Research on PHC delivery at the population level highlights two persistent problems: (a) inverse care (i.e., those who are most marginalized and have the greatest health problems have the least access to care); and (b) fragmentation and under-resourcing of care for marginalized populations, even in high income countries [1]. There are significant gaps in knowledge concerning: how to make services as responsive as possible for marginalized populations by more adequately addressing the health effects of structural inequities; how to make PHC services more socially relevant; and how to create policy and funding environments to support these aims [1, 16, 17]. Initiatives that focus solely on changing the knowledge or practices of individual practitioners are likely to have limited success unless they also consider the organizational contexts in which practitioners provide health care. However, the inherent complexity involved in developing and evaluating organizational-level interventions to promote equity-oriented care presents many challenges.

The purpose of this paper is to provide an overview of an innovative multi-component, organizational-level intervention designed to enhance the capacity of PHC clinics to provide equity-oriented care, particularly for marginalized populations. The intervention, known as EQUIP (the short name for Research to Equip Primary Healthcare for Equity) is being implemented and evaluated in four diverse PHC clinics in Canada. These clinics are located in two of the most highly populated provinces in Canada (Ontario [ON] and British Columbia [BC]), and serve as case studies for the implementation and evaluation of the EQUIP intervention. The clinics provide a wide range of interdisciplinary team-based services to populations ranging from 1300 to 6000 clients per clinic. The majority of clients are significantly affected by structural inequities and many experience major challenges accessing care. Clients include, for example, people with chronic health problems, chronic mental health and/or substance use problems, those experiencing systemic racism and discrimination including Indigenous people and racialized new immigrants, and women experiencing various forms of violence.

We begin the paper with an overview of the EQUIP intervention, starting with the evidence and theory that provide the basis for the inter-related intervention components, and a discussion of the expected impacts. We then describe the methods we are using to understand the process of implementing the intervention across diverse settings and to examine its effects. We conclude with a brief discussion of the integrated knowledge translation and exchange (KTE) activities designed to link the knowledge gained from this study with knowledge users and decision-makers who have the potential to influence uptake of equity-oriented care in PHC settings. Our aim is not to provide detailed protocols for implementing and measuring the impacts of the EQUIP intervention. Rather, by providing an overview of the intervention and our approach to evaluation, we hope to illustrate the complexities involved in attempting to enhance the health care sector's capacity to provide equity-oriented care in “real world” clinical contexts.

## Design and methods: Overview of the EQUIP intervention

The EQUIP intervention was designed to enhance the capacity of PHC organizations to be as responsive as possible to the diverse needs of populations whose health is influenced by intersecting forms of structural inequities. Using a combination of staff education and practice facilitation to support practice and policy changes at the organizational level, EQUIP provides a flexible structure in which both the general content and processes of the intervention are tailored to fit with the most salient issues and priorities at each clinic.

### Grounding the EQUIP intervention in evidence and theory

The EQUIP intervention is both evidence based and theoretically informed. Although many bodies of knowledge have informed the development of this intervention, research and theory related to equity-oriented care and complexity theory are particularly central to the design.

**Fig. 1:**
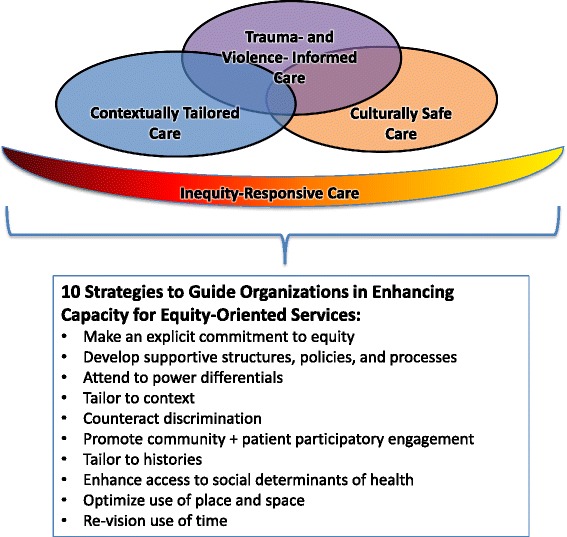
Key dimensions of equity-oriented PHC services

The content of the EQUIP intervention is based on an evolving conceptualization of equity-oriented care. Specifically, in previous research developed and conducted in partnership with PHC clinics and other organizations serving marginalized populations, we identified evidence- and theory-informed key dimensions of PHC services that position equity as an explicit goal [8, 18, 19]. Through the prior empirical work, we developed a framework identifying (a) four key dimensions of equity-oriented PHC services, which are particularly relevant when working with marginalized populations, and (b) following from those key dimensions, 10 strategies to guide organizations in enhancing their capacity for equity-oriented services, as detailed elsewhere [8]. Ongoing refinement of this framework led us to re-conceptualize inequity-responsive care as the overarching aim, and as foundational to supporting health and well-being through the provision of culturally safe care, trauma- and violence-informed care, and contextually tailored care (Fig. 1). Below, we briefly describe these key dimensions of equity-oriented services, which provide the basis for the EQUIP intervention components.

Increasingly, the concept of trauma is used to frame the health, social, and psychological effects of interpersonal violence [18, 20, 21–33]. Trauma-informed care (TIC) prioritizes the need to create an emotionally safe environment based on an understanding of the health effects of trauma. The insertion of violence into the notion of TIC is intentional to emphasize that (a) interpersonal and structural forms of violence (e.g., poverty, racism) intersect and (b) such forms of violence are often ongoing as well as historical, compounding the negative impacts. **Trauma- and violence-informed care (TVIC)** involves operating from the recognition that people impacted by social inequities often experience multiple forms of violence; the structural conditions of their lives often place them at greater risk of interpersonal violence, and of experiencing challenges in accessing supports to improve their physical and emotional safety. The emphasis on violence-informed care also mitigates the potential to locate ‘the problem’ of trauma primarily in the psyche of those who have experienced violence, rather than also in the acts of structural violence and the conditions that support those acts [34]. In contrast to more specialized ‘trauma therapy and trauma treatment’ such as psychotherapy, TVIC is a more general approach which aims to mitigate the potential harms and traumatizing effects of seeking health care or other services by creating safe and trusting environments [19, 21, 28–30, 35–37].

The concept of **cultural safety,** originally developed in New Zealand by Maori nurse-leaders, was intended to move nursing and health care practices beyond conventional cultural sensitivity training to more explicitly address inequitable power relations, institutionalized and interpersonal racism and other forms of discrimination, and the ongoing impacts of historical injustices on health and health care [38–41]. Cultural safety differs from the notion of cultural sensitivity, and aims to shift attention away from “cultural differences” as the source of the problem, and onto the culture of health care as the site for transformation [42–44]. Over the past two decades, cultural safety has been taken up internationally in diverse health care settings as a means of addressing persistent health and health care inequities [42–50]. In Canada and Australia, for example, cultural safety is often featured as an essential element of health care involving Indigenous and non-Indigenous people, and in New Zealand, is legislated as a basic requirement of nursing and medical professional education [51].

**Contextually tailored care** expands the notion of client-centred care to include services that are explicitly tailored to the populations served and to local contexts. This includes tailoring practices and/or organizational policies and clinical guidelines/protocols to optimally address the most pressing needs of local populations, and the social and community realities that often shift depending on local politics, epidemiological trends, etc. At the organizational level, contextually tailored care requires understanding the local community and context, along with mechanisms for developing and updating this knowledge continuously.

Complexity theory, an emerging approach in a range of disciplines including population health [52–55], was used to inform our thinking about the intervention structure and implementation process. Complexity theory is particularly useful for understanding health care organizations as complex adaptive systems with unique histories, structures, ways of operating, and community and funding contexts that shape how the intervention is taken up and its impacts. Rather than conceptualizing interventions with a ‘one-size-fits-all’ approach [56], or a tightly controlled design using standardized interventions [57], Hawe and others recommend that researchers: (a) expand their notions of interventions to make them adaptive; (b) broaden definitions of intervention success; (c) allow for strategic redirection during implementation; and (d) expand understandings of the effects on health outcomes [52–54, 58]. Complexity theory, therefore, challenges researchers to design interventions that can be standardized in terms of the overall purpose, and tailored to meet the needs of the different contexts in which they are implemented without compromising intervention integrity [52, 53]. This redefinition of standardization has the potential to increase the effectiveness of the intervention by improving the *fit* between the intervention and the local cultural or social environment in which it is implemented.

### EQUIP intervention components

The EQUIP intervention aims to enhance capacity for equity-oriented care at the staff level (i.e., knowledge, confidence and practices), and at the organizational level (i.e., policy and operations). The EQUIP intervention is novel in its dual focus on:

- Staff education: using standardized educational models and integration strategies to enhance staff knowledge, attitudes and practices related to equity-oriented care in general, and cultural safety, and trauma- and violence-informed care in particular, and;
- Organizational integration and tailoring: using a participatory approach, practice facilitation, and catalyst grants to foster shifts in organizational structures, practices and policies to enhance the capacity to deliver equity-oriented care, improve processes of care, and shift key client outcomes.

**Fig. 2:**
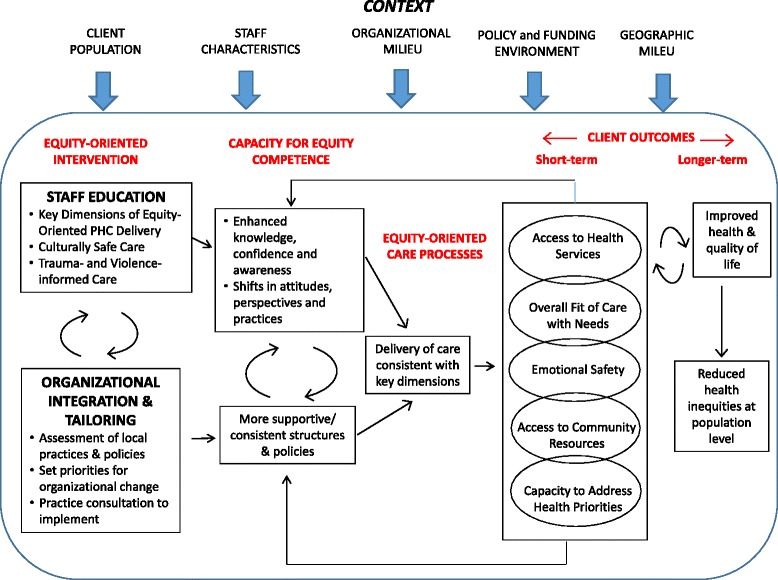
EQUIP intervention theory

The theoretical model of the intervention is depicted in Fig. 2.

Based on a view of health care organizations as complex adaptive systems, the EQUIP intervention was developed using core concepts and strategies that can be tailored to local contexts [52, 54, 58]. Given the goals of the intervention, and the fact that implementation depends on support and participation of administrators and staff working in busy clinical settings, we planned the intervention so that it could be delivered in phases over a 12 to 24 month timeframe.

Given the focus on change at both staff and organizational levels, we designed intervention activities to be appropriate for all staff, regardless of their specific roles, by taking varied learning styles and expertise into account. Within the intervention, three inter-related standardized educational components, described below, were delivered in ways which were tailored to local contexts and specific populations served, enhancing the relevance of the intervention [52, 54, 58]. A practice consultant, trained by the research team, facilitated implementation of the intervention within each setting by delivering the specific components, working with staff to integrate learning from the educational components, and serving as an ongoing resource for staff and the organizations as they worked through the intervention activities. Consistent with the participatory and integrated knowledge translation approaches used in this research program, our practice partners actively participated in developing the intervention and the study design, and provided ongoing input into the realities of implementing this kind of intervention.

### Staff education - Three components

Health professional education has only recently included concepts from a health equity and population health approach, and rarely addresses the health consequences of violence, trauma, discrimination and racism [59]. As such, education is one pathway for building staff capacity within organizations – to better understand and respond more effectively to people impacted by structural inequities and structural violence. While didactic educational strategies by themselves tend to not be drivers of behaviour change, the tailored educational and integration strategies offered through EQUIP created catalysts for change in each of the sites [60, 61].

#### Component 1: Orientation to key dimensions of equity-oriented PHC services

Two-hour workshops were offered to all staff at each site to provide an overview of the key dimensions of equity-oriented PHC and 10 strategies for enhancing capacity for equity-oriented services as shown in Fig. 1 above. Using interactive learning activities, these sessions drew on staff experience and knowledge. Throughout the intervention, the practice consultant was available to revisit and discuss this content with staff with a view to integrating these ideas in relation to Components 2 and 3 specific to each setting.

#### Component 2: Orientation to cultural safety

The primary content of Component 2 was an existing online, 8-hour, self-directed program known in Canada as the Indigenous Cultural Competency (ICC) program^3^. It includes interactive activities facilitated online by skilled adult educators and features content and cases involving Indigenous people in the Canadian context. The theoretical foundations of the ICC program include anti-racist pedagogy, critical race theory, and transformative learning principles, all of which align with cultural safety, which is also explicitly used. The goals of the ICC program are to stimulate positive changes in knowledge and attitudes about Indigenous people that are also generalizable across diverse cultural and social groups, and heightened sensitivity to racism and stereotyping generally. To foster integration of the ICC program’s content in relation to the diverse local populations served by the four PHC clinics, the practice consultant facilitated ‘integration sessions’ with staff at each clinic to consider implications for enacting cultural safety and countering discrimination in their local contexts. These sessions created opportunities to extend beyond what is often taken up as superficial attention to cultural practices in more standard cultural sensitivity and cultural competence training programs.

#### Component 3: Orientation to trauma- and violence-informed care

The third component of staff education addressed key approaches to TVIC, as defined above. Building on existing curriculum on trauma-informed practice developed in Canada [62, 63], the EQUIP TVIC curriculum was developed to focus explicitly on: (a) ongoing structural and interpersonal violence, as well as historical and intergenerational trauma; (b) how these factors intersect with poverty, racism, chronic pain, mental health problems and substance use, especially in the context of PHC; and (c) how action is required at all levels including practices, organizational approaches and policy. The TVIC training included eight hours of face-to-face workshop-style content with opportunities for small-group discussion and applied learning via case studies. Congruent with complexity theory, the training included common, standardized training modules, with discussions and clinical examples tailored to the key priorities identified by staff at each clinic.

### Organizational integration and tailoring (OIT) of intervention

The EQUIP intervention is grounded in the assumption that changes in staff knowledge, attitudes and practices are unlikely to result in significant shifts in equity-oriented processes of care unless attention is directed toward: (a) supporting staff to integrate learning from each of the three components in the context of practice (*integration*); and (b) creating locally relevant structures and processes within each organization to support such change (*tailoring)*. As described above, the process of integrating personal learning into practice was initiated during staff education and continued throughout implementation of the intervention. To facilitate the OIT process, each site received a $10,000 catalyst grant to be used within a 12-month period. These grants provided the impetus for clinics to identify and address short-term goals and strategies to further the delivery of equity-oriented care, recognizing that change is an ongoing process that will continue to evolve beyond the study’s time parameters.

Within each site, the OIT processes were initiated by the clinical administrative leaders/managers in consultation with experienced clinicians and staff who engaged in the following steps:

1. *Assessing the strengths, weaknesses and opportunities for enhancing equity-oriented PHC* on three levels: (a) their individual interactions with clients; (b) team processes and practices, including types of programs offered and/or ways of delivering specific programs (e.g., communication, documentation, managing referrals); and (c) organizational structures and policies that direct how services are delivered (e.g., policies about missed appointments, waiting lists, outreach activities, physical set up);
2. *Reviewing clinic profiles prepared by the EQUIP team,* including selected health and social status indicators (e.g., trauma symptoms, depression symptoms, languages spoken, income) for their clients compared to local, regional and national population norms to identify foci for change;
3. *Selecting 3–5 priorities for organizational change* based on the assessment;
4. *Developing a detailed plan for addressing each priority*, specifying goals, strategies, timelines and responsibilities, and a proposed budget for the catalyst grant;
5. *Implementing and evaluating* the proposed changes within a 12-month period.

OIT activities at the four PHC clinics include, for example: adapting the waiting room environment to be more welcoming for families caring for young children; developing and integrating harm reduction strategies into clinical programming and care; developing supports to address vicarious trauma experienced by staff when responding to the needs of clients experiencing violence; and expanding the approaches used to assess and respond to clients’ experiences of chronic pain, among others. In each site, the practice consultant was available to help problem-solve issues during implementation, reinforce the principles of equity-oriented care, and assist staff to evaluate the impact of their efforts and adjust the plan as needed.

### Expected impacts of the intervention

Figure 2, above, illustrates the proposed ways in which the EQUIP intervention could theoretically lead to a reduction in health inequities at the population level. We propose that engagement in the EQUIP intervention may enhance staff knowledge, confidence and practices, and shift policies, structures and operations in the clinics to better align with the principles of equity-oriented care, leading to enhanced delivery of care. As care becomes more responsive to client priorities and preferences, positive short-term changes for clients may include: improved access to health services and community resources; enhanced emotional safety and sense of respect during health care encounters; increased capacity to seek help to address health priorities; and an improved overall fit of care with needs. Longer term, access to equity-oriented care may lead to improvements in overall health outcomes and quality of life.

Given that PHC is delivered within complex adaptive systems, we argue that multiple factors shape how equity-oriented PHC is taken up within organizations. These include: (a) the characteristics of the population; (b) the characteristics of the staff; (c) the organizational milieu, including formal and informal power structures, policies, and funding; (d) the political, policy and economic contexts, particularly government directives that affect health care delivery and factors influencing the broader determinants of health; and (e) the historical and geographic context, specifically, the physical location of organizations in varied rural and urban locations, and the social conditions linked to those locations. As we discuss below, the methods we are using to evaluate the implementation and impacts of the intervention permit us to pay close attention to these contextual factors.

## Evaluating the implementation and impact of the EQUIP intervention

Using a mixed methods, multiple case-study design, we are currently examining the impacts of the EQUIP intervention in enhancing staff knowledge, confidence, attitudes and practices; improving processes of care; shifting organizational policies and structures; and improving selected client outcomes. A multiple case study is a comprehensive research strategy useful in exploring, describing, explaining, and evaluating causal links in real world interventions that are too complex to be assessed by survey or experimental strategies alone [64, 65]. This research design is also useful for describing the process of implementing EQUIP, including how the context shapes uptake of this intervention in diverse PHC settings. Although we are assessing the impacts of the intervention on clients, staff and the organization, the PHC site is the primary unit of analysis. Drawing on multiple sources of quantitative and qualitative data, our goal is to generate a detailed understanding of each case and a more generalized understanding of commonalities across cases [65].

Within Canada’s publicly funded health care system, PHC is a provincial responsibility, resulting in diverse models of care and funding arrangements. Given the goal of understanding how context shapes the delivery and impact of EQUIP, we purposefully selected PHC clinics that would provide variation across five dimensions of context noted in the intervention theory (Fig. 2). Although the context of each clinic differs, the four clinics share some important features. Each has an explicit mandate to provide PHC and programming that is as accessible as possible given their local populations, and each offers team-based care from a mix of providers, such as primary care physicians, nurse practitioners, registered nurses, social workers, counsellors and other staff members, although the team composition varies by site. The clinics are located in diverse geographic areas including rural, regional and inner city settings, and have different histories (e.g., longstanding versus newer clinics). These important differences provide sufficiently rich case examples for both within-site and across-site analyses related to temporal changes in outcomes, and the influence of context on processes and outcomes. This will allow us to draw inferences regarding common and site-specific factors shaping the implementation and impact across the four sites.

### Quantitative assessments of impact of the intervention

Quantitative assessments of the impact of the EQUIP intervention on staff are being conducted at three points in time (at baseline, midway through the intervention, and following the completion of OIT) using an online survey designed to assess knowledge, attitudes and confidence in enacting practices related to the three components of staff education. We are examining changes over time in staff knowledge, attitudes, confidence and practices using statistical approaches appropriate to the level of measurement.

Quantitative assessments of temporal changes in processes of care and client outcomes are being conducted using data collected through a client survey at four points in time (at baseline, early in the intervention, midway through the intervention, and following). At baseline, a sample of 120–160 clients was recruited from each site, comprising a longitudinal cohort of 567 clients followed over 2.5 years. Clients were eligible to participate based on the following inclusion criteria: at least 18 years of age, able to understand and speak English, had made at least three visits to one of the clinics in the past 12 months, and intended to continue accessing services for the two years following recruitment. Recruitment occurred by inviting all clients who met the inclusion criteria, and who came to the clinic on purposively selected days, to participate. To enhance representativeness, both clients who had scheduled appointments and those who “dropped in” were invited to participate.

The structured survey is comprised of investigator-developed survey items, standardized self-report measures and open-ended questions designed to measure the main concepts in our intervention theory, namely: (a) health care experiences, particularly perceptions of equity-oriented care; (b) short-term client outcomes, such as access to health services or community resources, capacity to address their health priorities, and overall ‘fit’ of services; and (c) longer-term outcomes including health status (e.g., chronic pain, depression, symptoms of post-traumatic stress) and quality of life. Demographic information (e.g., age, gender, employment status, financial strain, Indigenous identity) was also collected. To enhance retention, we maintained contact with participants on a regular basis between the waves of data collection, and offered honoraria to acknowledge the time and effort required to complete the client interviews. Our retention rate after four waves of data collection was 77 % across the four clinics.

Our initial analysis will focus on characterizing any changes in the main concepts identified in the intervention theory in Fig. 2, using statistical modelling techniques to allow comparisons of changes within and across clinic sites, and to model in predictors of such change. We will focus initially on changes in equity-oriented processes of care and short-term client outcomes, since we expect that these outcomes are more likely to change in the relatively short study timeframe, while changes in health status and quality of life will take longer to achieve. Next, we will examine the mechanisms of change suggested in our intervention theory by testing a series of causal models. Consistent with our case study design, we expect to test both general models that apply across clinics, and clinic-specific models based on the focus of their OIT goals and the specific context. For example, in a clinic where OIT focused on introducing improved guidelines for the management of chronic pain, we may test a model linking: (a) changes in client perceptions of equity-oriented care; (b) overall fit of care with needs (a short-term outcome); and (c) level of disabling chronic pain (a health outcome). The specific models to be tested will be informed by our theoretical framework and earlier analyses of client data, as well as emerging insights gained through the qualitative exploration described next. Given the sample size, clinic-specific analyses will incorporate a limited number of variables in order to ensure adequate statistical power.

### Qualitative exploration of processes of change and contextual influences

Qualitative research methods are ideally suited to studying both the process and impact of implementing the EQUIP intervention at each site, including how diverse contexts shape the uptake of the intervention. We are: (a) conducting in-depth, open-ended interviews with staff and administrative leaders; (b) conducting general observations of the milieu at each setting, and more detailed observations of staff meetings recorded as fieldnotes; and (c) analyzing policy and contractual funding documents to consider how they both shape and are shaped by engagement with the intervention. The in-depth interviews focus on staff members’ experiences of engaging with the EQUIP intervention, including challenges and successes, and their perceptions of any effects on their practice, team processes, approaches to care, and organizational policies and structures. These methods of data collection are essential to the multiple case study design in order to generate both a detailed understanding of each case, and a more generalized understanding of commonalities that exist across cases. Particular attention will be paid to understanding which aspects of context best explain differences in the intervention’s impacts across the sites. The qualitative findings will also be essential to contextualize the quantitative analysis of temporal changes described above.

## Discussion and future directions

Our multiple case study design and use of complexity theory provides an ideal opportunity to study the contextual factors shaping the implementation, uptake and impact of a complex, tailored intervention within diverse PHC settings. As our analysis proceeds, findings related to the impact of the EQUIP intervention will provide evidence about the practice-level changes, and policy and funding contexts needed to enhance capacity to provide equity-oriented care for people who are most impacted by structural inequities and structural violence.

Integrated KTE activities cut across all aspects of our intervention research, and involve clinical leaders within the sites and knowledge users in policy-making positions collaborating in planning and delivering the intervention. Describing the site-specific contextual factors and decisions regarding how to tailor and implement the intervention becomes, *de facto*, the first step in an evolving intervention-specific KTE strategy. For example, identified enablers and challenges in each context are becoming ‘lessons learned’ about what works, what does not, and why. This is informing our understanding of how the intervention (or aspects of it) could be tailored in new jurisdictions. These integrated KTE activities are informing our understanding of the complex factors that may intersect to influence implementation and the possible impacts in new sites outside of this research context. Longer term, this analysis will enable thinking beyond the specific PHC sites to inform a more policy-oriented approach to equity-oriented interventions, and ultimately, equity-driven system transformations.

## Abbreviations

BC: British Columbia, Canada
ICC: Indigenous cultural competency
KTE: Knowledge translation and exchange
OIT: Organizational Integration and Tailoring
ON: Ontario, Canada
PHC: Primary health care
TIC: Trauma-informed care
TVIC: Trauma- and violence-informed care

## Footnotes

1 PHC is conceptualized as the principal vehicle for the delivery of health care at the most local level of a country’s health system. Primary care is one of PHC’s core services because it serves as the first point of entry, provides person-focused care for all but the most uncommon conditions, and integrates or co-ordinates care provided elsewhere.

2 In this research program, the terms ‘marginalization’ or ‘marginalized’ refer to the social, political and economic conditions that create structural, social and health inequities in Canada and other nations, versus a characteristic that can be attributable to any particular population or group.

3 The Indigenous Cultural Competency (ICC) program was developed in British Columbia (BC), Canada, by the Aboriginal Health Division of the BC Provincial Health Services Authority (PHSA), and has been adapted for use in other provinces across Canada, and in Australia. Further information about this program can be found at: http://www.culturalcompetency.ca/.
